# The importance of introducing artificial intelligence to the medical curriculum – assessing practitioners’ perspectives

**DOI:** 10.3325/cmj.2020.61.457

**Published:** 2020-10

**Authors:** Ivo Dumić-Čule, Tin Orešković, Boris Brkljačić, Mirjana Kujundžić Tiljak, Stjepan Orešković

**Affiliations:** 1 Children`s Hospital Srebrnjak, Zagreb, Croatia; 2 IBM, Chief Analytics Office, New York, NY, USA; 3 Department of Diagnostic and Interventional Radiology, University Hospital Dubrava, Zagreb, Croatia; 4 Department of Medical Statistics, Epidemiology and Medical Informatics, Andrija Štampar School of Public Health, University of Zagreb School of Medicine, Zagreb, Croatia; 5 Andrija Štampar School of Public Health, University of Zagreb School of Medicine, Zagreb, Croatia; 6 University of Zagreb School of Medicine, Zagreb, Croatia

## Abstract

**Aim:**

To assess the attitude about the importance of introducing education on artificial intelligence (AI) in medical schools’ curricula among physicians whose everyday job is significantly impacted by AI.

**Methods:**

An anonymous questionnaire was distributed at the national level in Croatia among radiologists and radiology residents practicing in primary, secondary, and tertiary health care institutions, both in the private and the public sectors. The overall response rate was 45% (144 of 321).

**Results:**

A large majority of participants – 89.6% (95% Agresti-Coull confidence interval 0.83-0.94) agreed on the need for education on AI to be included in medical curricula. Answers revealed a very high support across age groups and regardless of subspecialty area. A slightly higher support was present among physicians working in university hospitals compared with those in primary care centers, and among radiology residents compared with radiologists – but these estimated differences are uncertain, and the support levels were clearly high across the considered variables.

**Conclusion:**

Since medical students have previously been shown to support introducing education on AI, a growing literature argues the same for reasons here reviewed, and physicians practicing a highly relevant area (radiology) overwhelmingly agree, we conclude that medical schools should indeed take steps to keep pace with technological progress in medicine by including education on AI in their curricula, be it as part of existing or new courses.

Advancements in medical technology in the era of the fourth industrial revolution are led by artificial intelligence (AI) and machine learning, aimed to enable the 4P model of medicine: predictive, preventive, personalized, and participatory (1,2). Development of AI tools already aids certain processes in the practice of several medical professions, such as radiology (3), dermatology (4), ophthalmology (5), and pathology (6), where Food and Drug Administration-approved AI-based algorithms are used (7). Radiology, in particular, has undergone dramatic, revolutionary changes driven by technological innovations in the past – the relevant achievements in AI are the latest breakthrough poised to become a part of widespread everyday practice, with the aim to improve the efficiency and (broadly defined) accuracy of radiologists and accessibility to their services. Among the FDA-approved AI-based algorithms are some that have achieved impressive reliability in diagnosing specific conditions, with specificity and sensitivity comparable to those of human experts for in-practice applications (8).

Due to these technological developments, a growing literature has emerged on the attitudes toward AI in medicine. According to initial surveys, the perception of AI among radiologists ranged between acceptance with enthusiasm and skepticism for fears of being displaced by the technology (9). However, the thus-far registered AI tools aim to aid radiologists in performing their duties, not to replace them. Perhaps in light of the increased popular awareness of this fact, recent research suggests that most radiologists strive to be included in AI education and research; that, on average, they would be willing to help in developing AI tools; and that they generally have a favorable attitude toward AI (10). An important factor in the development of these attitudes is education on AI, which is nowadays offered by major radiological associations as part of continuing medical education (11).

Only a few studies have so far explored attitudes about AI among medical students and their opinion on the importance of introducing AI-specific components as a standard part of medical education. A multicenter survey among medical students showed an absence of fear of being supplanted by AI in the future. More than two-thirds of respondents agreed on the need for AI to become a part of medical training (12). Another study concerns medical students’ attitudes toward AI, based on a survey at 19 medical schools in the UK (13). A majority (88.8%) of the students anticipated AI training would be beneficial for their future careers and believed it should be a part of mandatory curricula. Most respondents emphasized (89.6%), however, that with the state of their knowledge at the time of answering the question they would not feel capable to work with AI upon graduation.

The importance of including medical informatics into medical curricula has been widely discussed in the last few decades (14). Medical students show an awareness about the role of medical informatics in their future profession, with respect to supporting their professional work with patients, as well as in research (14). In certain medical curricula, medical informatics is comprised of two mandatory subjects, the first aims to provide essential knowledge at the start of one’s medical training, while the second is a more detailed upgrade, in the last year of the medical curriculum (15). Additionally, postgraduate programs sometimes offer various elective medical informatics subjects (15,16). Ensuring clinical data are recorded in a structured format appropriate for clinical tasks, communication with patients and colleagues, and for epidemiological purposes is one of the goals of medical informatics education and a prerequisite for implementing AI technologies. Another existing pillar essential for education on AI in medicine is the often-mandatory introduction to medical (bio)statistics course. Any additional parts of a medical curriculum focusing on AI in medicine should build on these natural foundations, as part of the same courses or as an independent one.

As the tide of AI in everyday medical practice is rising and the awareness of the need for appropriate education has already been explored among medical students, we conducted a brief national survey to assess the opinions of radiologists and radiology residents on the need for AI education in medical schools’ curricula. To the best of our knowledge, this is the first survey exploring attitudes about medical education among physicians whose profession is already significantly influenced by AI. As such, they are arguably in a still better position to judge the need for such education, since their judgment is, presumably, based not only on their expectations about the everyday practice in their profession but also on their experience of this practice – whether it has already been impacted by AI or is yet to be.

## MATERIALS AND METHODS

An anonymous electronic survey (Google Forms, Google LLC) was distributed among radiologists and radiology residents practicing in primary, secondary, and tertiary health care institutions at the national level in Croatia, both in the private and the public sectors. The survey was distributed via email to 321 members of the Croatian Radiology Society. If no answer was submitted within 30 days, a reminder was sent and, if an answer was not received within the following 30 days, a second reminder was sent; 90 days after the first email was received, the survey was closed. The introductory message contained no background information on the subject matter, nor any references to suggested reading material, to avoid biasing the responses. A total of 144 answers were collected. Among the respondents, 62.5% were radiologists and 37.5% radiology residents. A total of 54.86% of the respondents were female. Since the main aim was to estimate the overall level of support among radiologists and radiology residents, the finding was the sample proportion and accompanying appropriate type of 95% confidence interval of the total positive (“yes”) answers to the question: “Do you believe that education on AI should be part of medical schools’ curricula?”

To explore whether this attitude varies with characteristics hypothesized to be possibly relevant, the following logistic regression model was fit to the data:



Here the *Pr(y_i_ = 1)* stands for estimated probability of the radiologist/radiology resident responding positively, ie, agreeing that education on AI should indeed be part of medical schools’ curricula; *training* is a binary variable indicating whether the respondent is a radiologist or a radiology resident; *gender* indicates gender (with “male” as the baseline); and *institution_type* is a vector of four binary variables indicating which of the distinct medical institution types the respondent is employed at: primary care facility (baseline category), general hospital, university hospital, or private practice. These associations are also explored graphically, with no lesser importance given to this mode of exploration. Two more associations were added at this stage: *age_group* is a vector of binary age categories [20-29, 30-39, 40-49, 50-59, 60-69, >70], not included in the logistic regression due to a concern of collinearity when *training* was already included; and *subspecialty* – a vector of eight binary variables indicating the radiologists’ area of expertise, which was not included in the logistic regression model in the interest of parsimony.

## RESULTS

**Figure 1:**
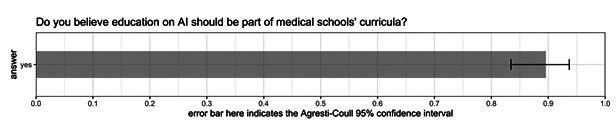
Answer to the question “Do you believe education on AI should be part of medical schools` curricula” for the whole sample.

The overall response rate was 45% (144 of 321). The percentage of respondents who stated that education on AI should be part of medical schools’ curricula was a very high 89.6% (Figure 1). Since the negative responses were relatively few, the Agresti-Coull approximations were chosen to compute the 95% confidence intervals: 0.83-0.94 (the Wilson score 95% approximation interval produces the same range). That a majority of respondents would support an introduction of education on AI is not surprising, but this rate is substantially higher even than the rates reported in the above-discussed studies: it suggests that practitioners recognize the need even more clearly than students (in an above-discussed student survey, 71% percent supported introducing AI education). This is the main finding of our survey.

Since the logistic function is steepest at its center, a simple interpretation of the independent variables’ coefficients is to find the logistic function’s derivative at this central point, which is the upper bound for the difference in the estimated *Pr(y_i_ = 1)* associated with a one-unit difference in the independent variable: at this point the value is ¼ of the estimated coefficient (17). Compared with radiologists – assuming the other variables hold the same value for radiologists – being a radiology resident was associated with up to a ~ 16% increase in the estimated probability of answering affirmatively, but the standard error makes the association indistinguishable from 0 when generalizing (Table 1). Similarly, considering the estimate and the standard error, there was no significant difference in how probable men and women were to think AI should be part of the curriculum. Further, compared with working at a primary care facility, working at any of the three remaining institution types was positively associated with answering affirmatively; however, the uncertainty of the estimates again prevents generalization beyond the sample. In short, the logistic regression yielded no evidence that the attitude varied widely across the included variables.

**Table 1:** Logistic regression, aimed to reveal any variation in response across other variables

	Dependent variable
	Should education of artificial intelligence be part of medical school`s curricula?
Training: resident	0.63 (0.62)*
Gender: female	-0.11 (0.57)
Institution type: general hospital	0.25 (0.91)
Institution type: university hospital	0.82 (0.88)
Institution type: private practice	16 (1602.1)
Intercept	1.44 (0.89)
Observations	144
Log likelihood	-46.28
Akaike information criterion	104.56

*standard error in parentheses.

**Figure 2:**
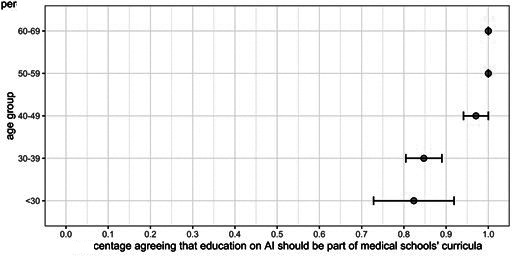
Variation in the response variable across age groups along with the standard deviations.

The remaining variable of most interest is certainly the *age_group* of the respondent (Figure 2). The direction of the association is somewhat surprising since younger practitioners might be expected to be more enthusiastic supporters of introducing new elements to medical schools’ curricula – this finding perhaps suggests that the positive relation between the attitude and being a resident is not merely due to age. However, given the uncertainty of the estimates, we resist reading too much into the patterns and take from this simply that the support is very high across age groups.

**Figure 3:**
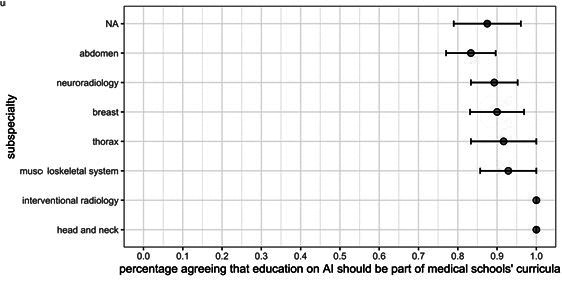
Variation in the response variable across subspecialties along with the standard deviations.

Much like across the rest of the variables (the rest are included in the model), respondents held a high level of support for introducing AI education regardless of their area of subspecialty (Figure 3).

**Figure 4:**
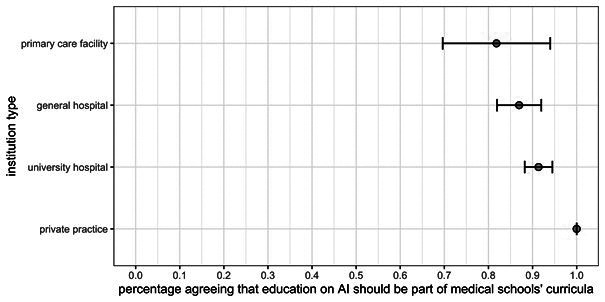
Variation in the response variable across institution type along with the standard deviations.

**Figure 5:**
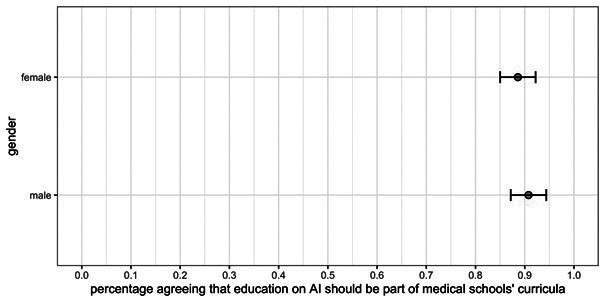
Variation in the response variable across gender along with the standard deviations.

The variation across institution types and genders is shown in Figures 4 and 5. Since the use of AI is more widespread in university hospitals than in primary care facilities and somewhat higher than in general hospitals, a level of support matching these patterns is in agreement with expectations, but the differences remain small and uncertain, while the percentage answering positively was again high across the categories.

Finally, although the response rate for the survey was relatively high (45%), we considered using multilevel logistic regression and poststratification to adjust for any known differences in independent variables between the sample and the population and how these might be differently associated with the attitude about education. In particular, this would involve adjustments based on the proportions of radiologists and radiology residents, as well as male and female respondents within the sample, compared with the proportions in the population, which are the population parameters known in Croatia. However, performing the first step, ie, the multilevel logistic regression, revealed that the estimates for how the outcome is associated with the four subcategories (eg, female radiology residents, male radiologist, etc) did not differ from the “main” non-varying intercept estimate. Adjusting the main estimate, hence, would not lead to a finding different from the one already reported: that ~ 89% (CI 0.83-0.94) of radiology practitioners believed education on AI should be part of medical schools’ curricula. The distribution by subcategory known both for the sample and the population is reported in Table 2.

**Table 2:** Distribution by gender and career phase for the sample and the population

	Male		Female	
Sample	No.	%	No.	%
radiologist	38/144	26.39	52/144	36.11
radiology resident	27/144	18.75	27/144	18.75
Population				
radiologist	235/750	31.33	296/750	39.47
radiology resident	97/750	12.93	122/750	16.27

## DISCUSSION

Our study showed strong agreement among radiologists and radiology residents on the need for education on AI to be part of medical schools’ curricula. It should be noted that an intrinsic limitation of a study based on a voluntary survey is a potential non-response bias: that is, individuals choosing to participate may differ significantly in some trait associated with the outcome of interest, compared with those who do not choose to participate. For surveys of this kind, the primary candidate for such a trait is possibly higher enthusiasm about the topic, here about AI. Another source of such a concern may be that the survey was conducted entirely online, in an environment possibly more comfortable to those inclined toward the use of technology; however, the use of email is in 2020 a low bar and should hardly be expected to systematically exclude a significant number of active practitioners. Furthermore, arguments to the contrary are at least as plausible: the survey was also an opportunity to express skeptical or negative attitudes on the question central to this study, as well as on the remaining questions (to be analyzed separately) on the respondents’ confidence in the use of AI in medical practice. Most importantly, the response rate was relatively high, as was the number of participants, and the sample did not differ significantly from the population in the known traits (gender, training), so further statistical adjustments (via multilevel regression and poststratification) based on those variables were not necessary. These considerations support a confident interpretation of the finding.

The finding is significant because the respondents are arguably in a suitable position to provide an informed opinion based on the impact of AI on their profession and its future. Considering the increasingly common use of AI in health care, medical education lags by at least a step behind technological developments. This has not been without consequences: a lack of knowledge among health care professionals is at the least a partial cause of the resistance in the adoption of AI in everyday practice, which is primarily manifested through various liability issues (18,19). To overcome the undue portion of this resistance, physicians should receive proper education enabling them to understand the inputs to AI algorithms, the algorithms themselves, as well to appropriately interpret their output (20,21). Efforts should likewise be made within AI to make algorithms not only ever more accurate but also more interpretable (22). It is hard to believe medical students will be prepared for the seemingly inevitable AI era in medicine, or that widespread general knowledge on the topic can be achieved without its presence as a part of medical schools’ curricula. On the other hand, it is encouraging to see a high level of support for education on AI among radiology practitioners, matching the technological advances and their impact on the profession.

Despite, as this study shows, widespread recognition of the need for innovation in education by a set of highly relevant practitioners (and despite significant changes in health care practice), medical education did not undergo the necessary changes. It is still largely based on traditional curricula comprising of various courses demanding, mostly, memorization of biomedical and clinical facts (22). As described by the Accreditation Council for Graduating Medical Education, learning outcomes and competencies attained by completion of medical school are nowadays globally similar, with the main focus on exposure to an immense amount of medical information and learning how to apply them in patient care (23). With few exceptions, medical schools have so far failed to recognize the importance of teaching on new technologies – on the possibilities emerging and changes already occurring with utilization of AI, mobile applications, wearable devices, and telemedicine – as part of their mandatory curricula (23).

The first policy proposal on how to implement AI in medical education was published two years ago by American Medical Association (AMA) and was followed by a number of initiatives at renowned institutions, which included, among others: opportunities for medical students to work in collaboration with data science experts, participate in summer courses dedicated to new technologies, and to be involved with relevant work at engineering labs (20,24). Notwithstanding the successful initiatives at certain institutions, systematic change in mandatory medical curricula has still not occurred. Several studies were conducted recently among medical students with the aim of assessing the opinion on and attitude toward AI using structured questionnaires (12,13). It was revealed that students received more information about AI from media than from university lectures. Only a small proportion of enrolled students underwent some form of AI education, but none of them received it as a part of their university curricula. However, more knowledgeable students were shown to be more open to working with AI-powered technology. The last example of a comprehensive and groundbreaking transformation of medical education was the Flexner Report (made by Flexner in collaboration with AMA) of 1910. Flexner reviewed all medical schools in the US and Canada and was focused on such criteria as admission standards, physical facilities, laboratory equipment, and instruction by physician-scientists. Following a screening of the educational system, Flexner recommended closing schools with poor standards and established the biomedical model as the standard for medical training (25). The age of AI in medicine calls for some innovation in the medical curriculum.

Following the studies exploring attitudes among medical students, we aimed to reveal the opinion of radiologists and radiology residents, whose everyday job already is or will be significantly impacted with AI, about the need for proper education at medical school. We found that radiologists and radiology residents overwhelmingly believed that AI education should be a part of mandatory medical school curricula, and seemed to believe so irrespective of their age, training, gender, workplace, and area of subspecialty. Since radiology is one of the several areas thus far most impacted by AI innovation, our results are arguably both (i) generalizable to the other similarly impacted areas (dermatology, ophthalmology, pathology), and (ii) serve as a look into the future attitudes to be expected among practitioners of other fields yet to be significantly influenced by AI. Most importantly, it seems clear that the dominant opinion among radiologists and radiology residents is that education on AI should be introduced to the medical curriculum. The finding suggests a positive attitude even more prevalent than has previously been found among medical students, albeit not in the very same context. Since medical students and practicing physicians of a representative, relevant area agree about the need to introduce AI in revised medical school curricula, while the growing significant literature argues the same for numerous reasons, there seems to be no excuse for medical schools not to take a step forward and try to keep pace with technological progress in medicine and look for appropriate ways to incorporate education on AI into existing courses or independently. Further work, beyond the scope of this analysis, should be devoted to the details of efficiently and appropriately incorporating such education.
